# ‘People don’t understand what we go through!’: Caregiver views on South Africa’s care dependency grant

**DOI:** 10.4102/ajod.v12i0.1114

**Published:** 2023-02-20

**Authors:** Zara Trafford

**Affiliations:** 1 Department of Psychology, Faculty of Arts and Social Sciences, Stellenbosch University, Stellenbosch, South Africa

**Keywords:** Care dependency grant, social assistance, caregivers, children with disabilities, South Africa, qualitative research

## Abstract

**Background:**

Caregivers are under enormous pressure in trying to provide for the needs of their children with disabilities in South Africa. The care dependency grant (CDG), an unconditional cash transfer, is the primary state-subsidised intervention for the social protection of low-income caregivers of children with disabilities.

**Objectives:**

The primary objective of this substudy, within a larger multistakeholder qualitative project, was to investigate caregiver perspectives on CDG assessment and application, their beliefs about the purpose of the CDG and how they actually used these funds.

**Methods:**

Data for this qualitative research included in-depth individual interviews and one focus group discussion. Six low-income caregivers who were current or previous CDG beneficiaries participated. Deductive thematic analysis was conducted using codes related to the objectives.

**Results:**

Access to the CDG was usually too late and over-complicated. Caregivers were grateful for the CDG but it was insufficient to cover the costs of care, in the context of high unemployment and weaknesses in complementary social services. Pressure on these caregivers was intensified by criticism in their social environments and a lack of respite care.

**Conclusion:**

Caregivers need service providers to be better trained and for systems of referral to available social services to be strengthened. The whole of society ought also to be targeted for increased social inclusion facilitated by improvements in understandings of the lived experience and cost of disability.

**Contribution:**

The rapid time from data collection to write-up of this study will aid in building the evidence base on the CDG, an urgent priority for South Africa’s journey towards comprehensive social protection.

## Introduction

The care dependency grant (CDG) is an unconditional cash transfer available to the primary care giver of a child with disabilities under South Africa’s social assistance programme. These children and their households are ‘the most economically vulnerable’ (UNICEF/DSD 2015:11) in the country, partly because of the high direct and indirect costs associated with disability (Hanass-Hancock et al. 2017). Direct costs include those that are specific to impairment (such as assistive devices, therapeutic services, accessible transport and caregiving), while indirect costs are those related to a loss of income or opportunity because of the demands of caregiving when appropriate child care is too expensive, inadequate or absent (Banks, Kuper & Polack 2017; Banks et al. 2021; White et al. 2018). In the context of extremely high unemployment rates and little respite or educational care for poorer children with disabilities in South Africa (SA), caregivers often have to leave work to care for their children, as reported since the early 2000s (De Koker, De Waal & Vorster 2006; Delany et al. 2005; Duma, Tshabalala & Mji 2021; Letsie 2016; Makwela & Smit 2022; Saloojee et al. 2007). According to international and regional conventions and local commitments to their civil rights, caregivers of children with disabilities in SA should have access to a wide range of interventions for social protection, including adequate and appropriate education and healthcare, assistive devices and spaces for play and recreation (Trafford et al. 2021). However, appropriate public services are generally insufficient, inaccessible or unavailable for many families, especially those who care for children with disabilities (Modula 2022; Philpott & Muthukrishna 2019; Tigere & Makhubele 2019). In practice, then, the CDG is SA’s key (and often the only) intervention for the social protection of children with disabilities and their families.

Numerous studies have examined the other cash transfers available under the SA social assistance system, a valuable knowledge base that captures a diverse range of perspectives and uses both quantitative and qualitative data sets (Adato, Devereux & Sabates-Wheeler 2016; Granlund & Hochfeld 2020; Hajdu et al. 2020; Kelly 2017, 2019; Oyenubi 2021; Patel, Hochfeld & Chiba 2019; Schneider et al. 2011; Zembe-Mkabile et al. 2015). However, studies of social assistance available to people with disabilities or children in SA often exclude the CDG from deeper analyses because of its low beneficiary numbers. As such, there is only a limited literature that is specifically focused on the CDG (De Koker et al. 2006; Delany et al. 2005; Dimhairo 2013; Khumalo 2020; Letsie 2016), some of which is now out of date or based only on desktop reviews. More regular and diverse investigative work is needed in this area (Tigere & Makhubele 2019). To contribute to growing this evidence base, a multistakeholder qualitative project focused specifically on the CDG was conducted. As one of a range of stakeholders, this section of the project gathered the perspectives of six primary caregivers of children with disabilities who were currently or had previously been in receipt of the CDG. This article describes and discusses grant recipients’ narratives about the processes and procedures involved in applying and being assessed for the CDG. It also shares how this group of caregivers made decisions about using the CDG under low-income constraints and explores the negative effects of a specific kind of community and relational scrutiny, previously reported in Gauteng province (Letsie 2016). The article concludes with a brief discussion of the idea that the ongoing deprioritisation of this group in SA may be partly because of their (not so) ‘benign neglect’ by government and in society. This deprioritisation could be perpetuated unintentionally in the coming years, as calls for a universal basic income increase. If the country truly aims to meet its commitments to the well-being of children with disabilities and their families, deeper and more sustained attention must be paid to the CDG.

### Background to the care dependency grant

The CDG is sometimes grouped with two other grants (the local term for cash transfers) that are available for the support of children in SA: the child support grant (CSG) and the foster care grant (FCG). However, the South African Social Security Agency (SASSA), which administers applications for and the distribution of all grants, classifies the CDG as a ‘disability-related grant’ (Trafford & Swartz 2021). The CDG is available to the primary care giver of a child who ‘requires and receives permanent care and support services’ because of their ‘physical or mental disability’ (South African Parliament 2020). The CDG is distributed monthly to beneficiaries, from the time of approval until their child with disabilities turns 18 (SASSA 2021). The grant is currently worth R1980.00 (USD120.00) per month, and there were 155 717 beneficiaries as of the end of September 2022 (SASSA 2022a). Initially instituted in 1993, there was a brief uptick in CDG beneficiary numbers in the early 2000s (De Koker et al. 2006; Delany et al. 2005), but this quickly slowed and access is considered to be falling short of meeting the population-level need (Philpott & Muthukrishna 2019; Redfern 2014). However, as South African childhood disability prevalence data are of questionable validity, it is difficult to estimate the scale of exclusion (Kidd et al. 2018; Philpott & McKenzie 2017). The other two disability-related grants include: (1) the adult disability grant (DG), which has the same value as the CDG and is for adults ages 18–60 who cannot participate in the labour market because of: (1) impairment, and (2) the grant-in-aid (GIA), which provides an additional amount of R480.00/USD29.00 per month to recipients of the DG, older person’s grant or war veteran’s grant (SASSA 2022b). The GIA is a contribution to the cost of a part-time carer for individuals who need regular support from another person because of physical or mental impairment.

To gain access to the CDG, caregivers must meet certain eligibility requirements that are described in the Social Assistance Act, instituted in 2004 and amended in 2008, 2010 and 2020 (South African Parliament 2004, 2020). Only caregivers whose income falls under the means test for the CDG are eligible, with the exception of foster parents, who can access the CDG regardless of income (DSD 2022). The means test threshold is defined by calculating the annual value of the CDG and multiplying this by 10, so using the current annual value of the CDG, an applicant’s income cannot exceed R237 600.00 per year (DSD 2022:43). If an applicant is single, the annual income of a single applicant is taken into account – if they are partnered, half of the annual income of an applicant *and* their spouse is taken into account (DSD 2022). Thus, the limit on average monthly earnings is R19 800.00, but this does not exclude much of the population, considering only 2% earn above this threshold and the national minimum wage is R3570.00 per month (Goldman et al. 2021). A more important measure for access to the CDG is thus whether the applicant’s child is considered eligible by a medical doctor, who must assess a child’s impairment(s) and recommend whether or not the grant should be awarded (cf. Trafford & Swartz 2022, for a more detailed discussion).

## Research methods and design

### Participant recruitment and inclusion under COVID-19

The overall research project for which these data were collected was designed as a qualitative ethnographic study. This section of the study was designed to serve as a companion to reports on the perspectives of frontline decision-makers (Trafford & Swartz 2022); bureaucratic administrators (Trafford & Swartz 2021); key informants from civil society, social work and academia; and policymakers. The intention was to conduct a deep investigation in a specific geographic area to explore the perceptions of various relevant role-players regarding the intended use and actual function of the CDG, as well as their related subjective experiences. However, because of the strict ethical constraints on in-person research imposed by coronavirus disease (COVID-19), the original study design was not possible and data collection had to be moved online. It was particularly difficult to connect with caregivers. It seemed that only being able to conduct research remotely might perpetuate the exclusion already experienced by economically poor caregivers of children with disabilities in SA, so this was delayed as long as possible and alternative options were explored. Revisions to South Africa’s *Protection of Personal Information Act* (POPIA) were an additional barrier, because organisations that serve or support caregivers could not provide direct contact details, even for caregivers who might have expressed an interest in participating. Thus, an ethically approved recruitment flyer was circulated among organisational networks in the Western Cape and beyond. Under the oppressive conditions of lockdowns and an economic depression, participation in research was a sizeable request and few responses were received. However, because of the historical and ongoing neglect of this group, it seemed important to include all of their voices, without focusing too much on narrow inclusion criteria. Arrangements were thus made (via WhatsApp or phone call) to speak with *all* respondents who had initiated contact, resulting in a sample of women from different provinces, some of whom had raised their children with disabilities in different decades.

### Participant characteristics

Participants were all women without disabilities, ranging from 31 to 63 years old (Table 1). All were now or had in the past been in receipt of the CDG and were the biological mothers of the children for whose care they received the CDG. The terms ‘child’ or ‘children’ in the Results and Discussion sections of this article is not used to imply that those who were over 18 at the time of interview were still children or to infantilise them. Instead, the term ‘child’ was used in light of the relationship in question, because all of the participant caregivers were also the actual parent of the disabled son or daughter about whom they were talking. All but one of the participants lived full-time with their disabled son or daughter. One caregiver (CG2) shared the grant money for and care of her disabled daughter with her own biological mother, the child’s grandmother. Caregivers’ sons and daughters ranged from 9 (born 2013) to 36 (born 1985) years old (Table 1). One respondent’s son had died at the age of 22 in 2019. Half of the mothers had not finished high school, but some had later pursued diplomas or nondegree qualifications related to disability advocacy work (Table 1). As the focus of this work was on exploring the subjective experiences of caregivers through semistructured conversations (as opposed to a structured survey), gathering detailed information about these caregivers’ incomes was not the aim. All participants were, however, living in underserved and economically poor areas. Only two were currently employed and in receipt of regular but low income; one was a domestic cleaner and the other a carer-to-carer trainer. In some instances, the household gained a small amount of additional income from the respondent’s male partner, but this was not always regular, and mothers could not necessarily access these funds for the care of their child with disabilities. Four of the participants lived in the Western Cape and two were based in Gauteng, provinces in southern and central SA, respectively.

**TABLE 1:** Participant characteristics and demographics.

Participant number	Data collection activity	Employment situation	Age	Ethnicity and location	Level of schooling	Number of children	Child’s disability	Age of disabled child	Partnered
CG1	IDI	Unsalaried, semi-retired	63	Mixed race South African, WC	Graduate diploma obtained later	5 (3 biological)	CP; ID	36 (b. 1985)	Y
CG2	FGD	Unemployed	31	Black South African, WC	Grade 12 (Matric)	3	Chromosomal difference; ID	11 (b. 2011)	Y
CG3	FGD	Self-employed (ECD centre) but unsalaried	46	Black South African, WC	Grade 12 (Matric)	3	ASD	14 (b. 2008)	Y
CG4	FGD	Employed in domestic labour	32	Black South African, WC	Grade 11	2	CP	9 (b. 2013)	Y
CG5	IDI	Volunteer with a small stipend	35	Black South African, Gauteng	Grade 12 (Matric)	3	CP; ID; visual impairment	13 (b. 2009)	Y
CG6	IDI	Employed (carer-to-carer trainer)	53	Black South African, Gauteng	Did not finish high school	2	CP	22 (b. 1997 – d. 2019)	N

IDI, in-depth interview; FGD, focus group discussion; ECD, early childhood development; WC, western cape; CP, cerebral palsy; ID, intellectual disability; ASD, autism spectrum disorder.

### Data collection and analysis

Data were collected between July 2021 and April 2022. Data collection included three semistructured in-depth individual interviews (IDIs), with lengths ranging from 1 h 30 m to 2 h 15 m. In-depth individual interviews were conducted via WhatsApp video call at a time that suited the participant. Mobile data bundles were provided to participants prior to our conversations. One semistructured focus group discussion (FGD) of 4 h 20 m was also conducted, with an additional three caregivers. During the focus group, the author sat in a room with a two meter distance between each person, on chairs that had been sanitised. Each person wore an N95 mask and used hand sanitiser repeatedly. Due to the aforementioned ethical constraints on in-person research mandated by the Stellenbosch University Research Ethics Committee for Social, Educational and Behavioural Research [REC: SBE], data collection was only conducted in English, as it was not possible to work with an interpreter. English was not the participants’ first language, but conversations to arrange times and to build rapport were conducted with each participant before and after data collection. All participants expressed themselves fluently, both verbally and in writing, and also confirmed that they were comfortable speaking in English on the informed consent forms they were provided with prior to data collection activities.

Although the author did not communicate in the participants’ first language, all participants commented that the time had gone quickly and that they had enjoyed and appreciated the opportunity to talk about their lives, indicating that this was a positive experience for them. In the single instance during the FGD where one participant was unsure of the English word for a concept she wanted to express, she asked one of the other participants, who translated from isiXhosa for her, suggesting that she felt comfortable enough to ask her peers if she was unsure of anything. The three FGD participants had shared transport to the venue and were already friendly by the time they arrived. They shared jokes and commiserated with one another. This contributed to a relaxed environment, in which deeply personal narratives were quickly and openly shared. The participants all joked with and teased the author too, suggesting that a comfortable space was co-created in which the power differentials were not erased but were, hopefully, minimised. It is not possible to be certain, but it is not clear that the presence of a translator would necessarily have made participants more comfortable, as the additional person in the room may also have been perceived as a silent or judgemental witness.

All data collection and verbatim transcription were conducted by the author. Transcription served as initial data familiarisation, and repeated analytical reviews of the written transcripts allowed for the identification of common themes. Themes were discussed with a senior colleague, who also reviewed the penultimate and final drafts of this article. Analysis for this article was primarily deductive, and codes were drawn from questions relating to the original research project objectives. These codes were applied to IDI and FGD data. The results presented here focus only on the data that corresponded with these codes, which included eligibility rules for the CDG; caregivers’ experiences of the process of gaining access to and receiving the CDG; their beliefs about the purpose of the CDG; and its actual uses in their households. Forthcoming publications will provide additional insights into their experiences of life, caregiving and relationships with their children with disabilities.

### Ethical considerations

An application for full ethical approval was made to the REC: SBE, and ethical consent was received on 28 November 2019 (reference number PSY-2019-13097) and renewed annually. Procedures for participation and protection were in accordance with the ethical standards of the REC: SBE and with the 1964 Helsinki Declaration and its later amendments. As individual interviews were conducted via WhatsApp, specific permissions (including data safety and storage procedures) were sought and approved. Written and verbal informed consent was obtained from each participant. Each participant was also given a copy of the informed consent form to keep. Important consent issues were reiterated at the beginning and the end of each activity. In the focus group, it was emphasised repeatedly that although any quotes or data shared in this write-up would be anonymous, it was possible that other participants could breach confidentiality. Participants were thus urged to protect one other, so that they could share freely in the group, but not to feel obliged to answer any questions or to share anything that was so personal that they would feel vulnerable. Only the author has had access to the data set, which is stored on password-protected cloud storage and backed up to external storage. All the data have been anonymised using alphanumeric codes based on the order of activities (CG1, CG2, etc.).

## Results

### Who should tell caregivers about the care dependency grant, who is eligible and when and how should they gain access?

Participants felt that caregivers should be instructed to apply for the CDG immediately upon receiving a relevant diagnosis or at birth, if the child’s disability was already known. For them, there was a direct link between diagnosis of a child’s disability and access to the grant:

‘[*I*]f you have a child with a disability … you are eligible for the grant. Because … your child has got a lot of diverse needs … that you’re going to need to attend to.’ (CG1, 63 years old, Western Cape)

‘I think [*the CDG is*] supposed to be given by the doctor when the doctor diagnoses your child. But they don’t do that. With my son… I applied for a grant… three years [*after*] knowing that he has autism, because no one ever told me that he’s supposed to get a grant.’ (CG3, 46 years old, Western Cape)

‘[*…When*] they discharged me [*after my baby’s birth*], they knew what the problem [*was … but*] I didn’t immediately get the care dependency grant. When I [*went to hospital for my child’s appointment later, a*] doctor said, “No, you should get the [*CDG*]” … [*But*] it took [*8*] months, [*and*] I had to go [*to the offices*] … 20 or 30 times!’ (CG2, 31 years old, Western Cape)

In contrast to what they felt was appropriate, participant caregivers had generally had a gap of months or years between their child’s diagnosis and their receipt of the CDG.

An IDI participant from Gauteng explained that after being referred by her treating doctor, who indicated that her child was eligible for the CDG, she had to make numerous trips back and forth between two doctors in order to eventually gain access. One mother in the FGD had been given a form that certified her son’s impairment (cerebral palsy [CP]) and was instructed by hospital doctors to apply for the CDG immediately after he was born. However, when she had first tried to apply, she was turned away in the Western Cape and again in the Eastern Cape by SASSA clerks, who appeared to be making this decision themselves rather than relying on the doctor’s assessment form:

‘[… *T*]he doctor said, “He won’t walk, [*SASSA is*] supposed to give him this grant.” But [*SASSA said*], “No, we want to see [*for*] ourselves if, really, this child … won’t walk” … [*in the*] Eastern Cape, they [*told*] me the same: “We are not sure if he is disabled, so we are going to give you … R270.00 [*i.e. the child support grant at the time*]” … my son [*only*] got his [*CDG*] … after four years.’ (CG4, 32 years old, Western Cape)

Another IDI participant reported the same problem:

‘[*P*]eople are really struggling … They’ve been told so many stories, “Go back to your doctor, he will hand you the letter.” [*But*] that is a process! … So people end up giving up … [*But*] you look at the family, [*and*] they really need [*the CDG*].’ (CG6, 53 years old, Gauteng)

Participants indicated that the onerous process of trying to access the CDG was ‘too much’ for some other parents of disabled children whom they knew. These parents often gave up and settled for the CSG, which is worth less than a quarter of the CDG and is utterly insufficient for meeting their child’s needs.

The same FGD participant who had previously said that she had been to SASSA offices ‘20 or 30 times’ also commented that the officials she had encountered had been uncaring and unresponsive. After walking 10 km to these offices every day, she had called a helpline:

‘[*T*]hose people [*at SASSA*] … don’t care … you want to get information about what’s happening with [*your*] child’s grant [*application but they say,*] “No, there’s nothing we can do” … [*Later, I heard*] on the news, “If you have a problem with government issues, call these offices.” When I called … the guy was shocked … one hour [*later*], I received a call [*from*] the manager [*of the same local SASSA office*] saying, “Please can you come to our offices tomorrow so we can meet?”’ (CG2, 31 years old, Western Cape)

This parent was subsequently back-paid for the long wait, but others may not be as lucky. Although the number of visits made to SASSA offices may have been exaggerated, this was clearly an arduous and exhausting process. Many caregivers may not have the capacity to be as persistent. Caregivers in this study expressed serious frustration with long waiting times, poor and dismissive treatment from SASSA officials and a lack of appropriate referrals during the process of application for a CDG.

Discussion on the topic of severity and its influence on access to the CDG was part of the semistructured interview guide used for data collection. In response to questions on this topic, one IDI participant responded that she knew that approval of the CDG was often linked with a *severe* diagnosis (cf. Trafford & Swartz 2022) but worried that many medical assessors were not well-informed enough about childhood disability to accurately gauge severity. This could result in delayed access or inability to access the CDG, to which she believed these children were entitled:

‘[*Usually, with CP*] it’s a yes [*to the CDG*] … [*but*] it depends on … severity… At the age of three years, some parents say… they’ve been told [*by SASSA*] that it’s just a matter of time – the child will be able to do this, the child will be able to do that… [*But*] at the end of the day, the child is still in level four [*and*] doesn’t move [*independently*]!’ (CG6, 53 years old, Gauteng)

This participant had both the lived experience of parenting a child with CP and the professional experience of being a carer-to-carer trainer working with disability specialists in an organisation focused on supporting parents of children with CP. She was frustrated that if a child was born with, for example, Level 4 CP and would likely never be able to complete activities of daily living on their own, ‘waiting to see if [they] will walk’ was a waste of time, during which parents could be receiving the CDG.

Participants were also asked to comment on SASSA’s recent shift towards allowing online applications in an effort to improve access. One parent who had been involved in a parent-led disability advocacy organisation since the 1990s was concerned that moving these services online would actually result in *more* exclusion. While reflecting on her work with low-income parents in the COVID-19 pandemic, during which meeting spaces rapidly moved online, she observed the following:

‘[… *I*]t is difficult for [*low-income parents*] to do online processes … How do we make it accessible for parents … the new way of working via all these devices and platforms? … we struggled [*during COVID-19 lockdowns*] to connect to our parents … you will schedule a meeting for 10 o’clock … you will jump on, get disconnected, jump on, get disconnected. Eventually, you start your meeting at 11:30 … And it depends where the parents are situated … Is it an area that is accessible? Is it a rural area? … This is the new normal, but there’s a lot of things that still need to [*change*].’ (CG1, 63 years old, Western Cape)

This participant worried that an intervention that was supposedly designed to improve access would not actually reach those who were most disadvantaged by poverty, rurality and infrastructural weaknesses. More comprehensive intersectoral and thoughtful planning needs to inform attempts to upgrade such systems, in the context of widely varying resource distribution and patterns of access.

### The purpose of the care dependency grant

When asked what they understood the purpose of the CDG to be, caregivers generally said that its main function was to replace the income of a primary care giver of a child with disabilities because parents often had to leave their jobs to care for their child full-time:

‘[*M*]ost parents … can’t go to work, to go and look for a job … because we have these kids [*to care for*].’ (CG2, 31 years old, Western Cape)

Being forced to leave a job was both economically and emotionally difficult, and caregivers wished there was more state support in place to help:

‘I was going to be promoted to … trainee manager. [*But my child*] had to go for an operation, for the [*holes in her*] heart … I told my manager, “I need to stay [*with my child in hospital, please*] transfer me,” [*but*] my manager said, “No, I can’t lose you” … I had to decide ok, let me just quit my job and quit that opportunity that was going to be so beautiful, going to be life-changing for me.’ (CG2, 31 years old, Western Cape)

‘I used to have a better salary, to afford my kids. But I had to stop working, and [*now I*] have an ECD that is not being funded by the government, so there’s no salary, no stipend, no nothing … we’re supposed to get [*some support*] to look after these kids, or maybe someone from the government [*who can*] help us [*look after our kids while we*] go and look for a job.’ (CG3, 46 years old, Western Cape)

One participant observed that the CDG was also aimed at improving equity, as it might assist some parents in bringing the potential of their child with disabilities for access up to the level available for children without disabilities:

‘In my understanding, the grant … is whereby our social care workers, our doctors, and our government are trying to meet the parents halfway, so that the child can have a better life like any other child.’ (CG6, 53 years old, Gauteng)

All participants commented on the strain of having to care for a child with specific needs without adequate social or state-sponsored respite care.

Caregivers whose children were under 18 were also concerned about what would happen financially when their children reached adulthood, as they all expected their child to continue needing support beyond that age:

‘[*W*]hen it’s coming to the time where a child is changing [*from*] the care dependency to a[*n adult*] disability [*grant*], I don’t understand why there must be months that he doesn’t get [*support*] … I don’t see even the need to apply, because [*I think*] they’re supposed to check the child’s age and change [*the grant they receive*] … automatically.’ (CG3, 46 years old, Western Cape)

These caregivers emphasised the long waiting times they had previously described and were worried that it would take a long time to regain access or that they might be refused, even though they did not expect their children to be able to find employment as adults.

### The care dependency grant: Everything and nothing

As beneficiaries can decide how they use their CDG funds, parents were asked how they made decisions about expenditure. Costs that were specific to their child with disabilities included those previously reported among low-income caregivers: transport, school fees, specialised food and clothing, assistive devices, Internet and paying people to provide care to their children. It was also clear that even when complementary supports such as subsidised medication were supposedly available, these were often inaccessible or inadequately planned:

‘[*T*]he nappies are expensive, the transport is expensive … I really respect whoever came up with that idea that … children with disabilities must get free medication … But … it depends on the kind of medication, because [*some*] medication is expensive, you have to go to the pharmacy to get those ones. [*But*] you are not working – how can you afford those medications?’ (CG6, 53 years old, Gauteng)

Similarly, although some schools were subsidised, these were scarce, too far away or insufficiently resourced to provide what they promised:

‘[*My child*] needs physio[*therapy*] … they say at the hospital, “Now that your son is at [*a special*] school, the school is supposed to have a physio[*therapist*].” [*But*] the school doesn’t! You end up being a bad parent if you’re going to fight [*with the school*] … [*So*] I have to pay the school fees, the transport [*and*] … I have to pay for him to get physio [*privately*].’ (CG4, 32 years old, Western Cape)

Evidently, the CDG was often depleted by paying out-of-pocket for services that ought to be publicly available or by seeking expensive private care. Caregivers were grateful for this income and felt that it was ‘very important’ (CG3, 46 years old, Western Cape), but all observed that it was not sufficient for combatting the enormous exclusion and expenses they faced.

One mother, who had been in receipt of the CDG for 12 years, commented that the CDG was ‘a drop in the ocean’ (CG5, 35 years old, Gauteng) in the face of her monthly costs. When asked how she made plans about using the CDG from month to month and if this ever changed, she described the complicated calculations and compromises she regularly had to make in trying to ensure her children’s needs were met:

‘[…*W*]e’ve missed a couple of doctor’s appointments … because she has grown [*so*] I can’t carry her anymore … if I hire transport – I don’t have a car obviously – they charge me 750 to a thousand [*rand*] … one pack [*of*] 30 [*nappies*] is 370 [*rand, and*] I buy 2–3 [*packs per month*] … If I bought two for a month then … toward month-end, I have to buy a pack of ten, so it can last up until I get my grant … [*the same*] 750 [*I might have used for transport to her appointments*] … would cover one month of nappies.’ (CG5, 35 years old, Gauteng)

In addition, while all caregivers felt that ‘the money that [our children] get … is only for their personal [needs]’ (CG3, 46 years old, Western Cape), as this idea was discussed further, caregivers explained that CDG funds often had to be used to support others in the household. When faced with the needs of their other children and minimal or no alternative sources of income, caregivers had no choice but to juggle their priorities and try their best to balance expenses, which sometimes meant that there was not enough money available to adequately meet the specific needs of their child with disabilities.

Finally, these mothers felt strongly about providing well for their children with disabilities but often expressed guilt that they might be doing this improperly or insufficiently, which was closely related to the lack of support and training available to poorer parents of children with disabilities, especially those with less common or misunderstood impairments. For example, one mother said that she would massage her child’s muscles when his body got ‘stiff’, but she worried and was ‘afraid … [*that*] maybe I’ll be too harsh’ (CG4, 32 years old, Western Cape), because she had not been given sufficient training. Another, whose teenage son was autistic, described the costs associated with neurodiversity. Although her son did not need some of the consumables commonly associated with childhood disabilities (such as incontinence products), other purchases (such as Internet access) were important for maintaining her son’s quality of life and his routine, often critical for autistic people. This caregiver also tussled with the difficult emotional experiences she had had with her son, who could be destructive and aggressive when he was unhappy. She felt that ‘if the government gave us some training when they diagnose the child, then maybe I would be able to control [*his*] tantrum[*s*]’ (CG3, 46 years old, Western Cape)

### Others’ scrutiny and invasive comments on caregivers’ parenting and receipt of the care dependency grant

Caregivers in receipt of the grant reported that they experienced enormous scrutiny from their communities regarding their parenting and financial choices in relation to their child with disabilities. Onlookers perceived recipients of the CDG as having ‘a lot of money’ (because most other parents in their neighbourhoods would only be able to access the smaller CSG) and were highly critical if it appeared that these caregivers were not providing appropriate or sufficient care to their children with disabilities:

‘[*People*] are talking … “Your son get[*s*] more money!” … [*but*] they don’t understand the situation … The other day the social worker came to my house to say, “This lady … has laid a complaint about you … She’s complaining that you always lock your child in the yard” … I explained … that no, my son [*has*] autism [*so*] when he [*is*] outside, we have to make sure that there’s someone [*with him*] … Really, people … don’t understand what we go through!’ (CG3, 46 years old, Western Cape)

‘They don’t understand that it’s because my son has more … needs than their child. They think maybe I’m special, or the government did me a favour to give me more money.’ (CG4, 32 years old, Western Cape)

These criticisms were apparently based more on onlookers’ own beliefs about what care *looked* like, rather than a proper understanding of the circumstances or the needs of the child in question.

The reactions of neighbours and family members to their receipt of the CDG placed additional emotional and economic pressure on caregivers:

‘[*O*]ne time … [*my child*] didn’t have enough clothes and it was towards winter. There was a sale [*at a shop where my mother*] has an account. I asked her, “Mom, can you help me out with … clothes?” And she was like, “[*Your child*] gets her grant … It’s more than enough.” I think that’s when I really stopped asking for help from [*my mother*].’ (CG5, 35 years old, Gauteng)

A few respondents indicated that for some people, the pressure to show others that they were looking after their children properly might mean that they used CDG funds for less useful purchases that they would not otherwise prioritise:

‘There is pressure … I’ve seen some parents buy expensive clothes for their kids … Because what they’re getting from the society is: “You earn a lot of money for your child and yet your child is dirty, or they’re wearing cheap clothes, or they don’t have shoes, or they don’t have fancy food” … [*they think*] you are chowing [i.e. using up] their money for your own needs and you’re not taking care of your child.’ (CG5, 35 years old, Gauteng)

Although caregivers in this study argued vehemently that parents of children without disabilities could not understand how different (and expensive) their parenting experiences were, they still felt pressure to show that they were good parents. For them too, this meant keeping their children clean and well-dressed, which may have been intensified or influenced by onlookers’ explicit judgments on these visible aspects of care. In the FGD, one mother spoke with great pride about treating her disabled child and her non-disabled child exactly the same as one another:

CG4, 32 years old, Western Cape: ‘I have two boys and they like clothes … I bought them pairs of Nike, so they are the same, because people like to say, “Ohh wow, [*your nondisabled child’s*] shoes are much better than [*your disabled child’s shoes*]!”’

CG2, 31 years old, Western Cape: ‘It’s irritating!’

CG4, 32 years old, Western Cape: ‘I say … “Don’t talk to me like that because these are my children and I like them equally!” … So I bought them the same shoes … And then they’ll say, “Ooh, you bought [*your disabled child*] these shoes but he can’t [*even*] walk?!”’

ZT: ‘[*So*] it’s never good enough?’

CG3, 46 years old, Western Cape: ‘Never!’

CG2, 31 years old, Western Cape: ‘Never!’

CG4, 32 years old, Western Cape: ‘Never *never*!’

This also led to some caregivers differentiating themselves from other parents of children with disabilities who they felt were less caring and attentive:

‘[*…O*]ur kids they are the cleanest, our kids they are the most beautiful kids … [*my child*] looks spot on. The teacher even sends me pictures … I’m happy because … there are people with a child with disability who cannot even bathe their kids … they will just leave their child looking anyhow.’ (CG2, 31 years old, Western Cape)

By the end of a long conversation on this topic, which was also explored with IDI participants, caregivers were unanimous that how they treat their children with disabilities was ‘never enough’ for onlookers, whose criticisms were often contradictory. These dynamics had implications for their sense of belonging in the community and, potentially, for caregivers’ use of CDG funds.

## Discussion

Early intervention is especially important for children with disabilities or chronically ill children, who may require more extensive health and therapeutic care in their early years (Kanji 2021; Moodley 2021; Sherry 2015; Storbeck & Moodley 2011). Access to this care can have dramatic effects on their long-term development and well-being. Some of the caregivers in the study reported on in this article had been directed to apply for a CDG by their child’s treating doctor, either at the point of diagnosis or during a subsequent healthcare appointment. However, as also reported by CDG beneficiary participants in Gauteng-based studies (Dimhairo 2013; Letsie 2016), all but one of the caregivers in this study spent months or even years attempting to gain access, despite being eligible for the CDG. Access tended to be *ad hoc* and reliant on chance meetings with specific individuals, rather than happening along a predictable pathway. Late access to the grant had constrained caregiver capacity to seek early intervention for their child with disabilities, which had an impact on their child’s physical or mental well-being and had also been emotionally painful for the parent. Some caregivers expressed concerns that they were not given enough information and did not understand their children’s disabilities well enough to support them properly, especially in the earlier years of their children’s lives. The pattern of late and unpredictable access to this critical support has previously been reported in Gauteng (Dimhairo 2013; Letsie 2016) and may be having a deep and as yet insufficiently documented effect on developmental progress and the well-being of both children with disabilities and their caregivers. The pressure on the caregivers in this study was compounded by a lack of acceptance from their social environments. This situation is currently not much changed since Swartz (2012:37) reported that ‘[*i*]gnorance, fear and anxiety, and lack of skill are major issues which affect how able-bodied people at all levels of society interact with disabled people’.

Caregivers commented specifically on the lack of clarity on the progress of their application and the severity thresholds governing approval and rejection for the CDG during their journey to access. They did not understand how two CDG applicants whose children had the same diagnosis could have different application outcomes. Because severity thresholds are not explicitly dictated by SASSA, different assessors may apply different thresholds, which can result in variable, uneven inclusion (see Kelly 2016b; Trafford & Swartz 2022, for these discussions on the DG and CDG, respectively). The result is that these processes feel unpredictable to those who most need to understand how to navigate the system and even, in fact, to those who are implementing the assessment system at the frontline. In addition, poor communication and dismissive treatment from SASSA officials and assessing doctors can make the process of accessing a CDG feel even more difficult for potential applicants. It is likely that focusing first on improving communication with and customer service for SASSA clients would yield more rapid positive results than the recent attempts to move applications online, which could actually serve to further exclude those most disadvantaged by structural inequality. Effective improvements to the social assistance system will require much deeper thought about what kinds of limitations already exist and how certain changes may intensify exclusion, even when this is unintentional.

As the use of the CDG is not explicitly dictated by SASSA and the cash transfer is unconditional, caregivers have some autonomy in choosing how they will spend these funds. This is positive because it allows families whose children have diverse or unexpected needs, as was the case with the parent of an autistic teenager in this study, to make their own financial decisions. However, because of inadequate or inaccessible public service provision, caregivers were regularly forced to spend out-of-pocket on expensive private transport, medications, special foods or other impairment-related products, which rapidly depleted their CDG funds. In addition, many of the mothers in this study had to leave their income-generating work and did not or could not obtain financial support from their children’s fathers. Thus, CDG funds were commonly used to support the needs of the whole household, as is common for all of the grants available under SA’s social assistance provisions (Granlund & Hochfeld 2020; Kelly 2016a; Kidd et al. 2018; Lloyd-Sherlock & Agrawal 2014). As the caregivers whose narratives are shared in this article were based in peri-urban areas in Gauteng and the Western Cape, the most well-resourced provinces in the country, the situation for caregivers in other provinces and rural contexts is likely to be even more difficult and isolating (Duma et al. 2021; Modula 2022). The revitalisation of and better linkage to existing services, or the initiation of new services to which these families are legally entitled, would greatly enhance the impact of the grant, allowing it to be more than just a basic survival mechanism.

At the very least, as a concession to respite care, there is a strong case for extending the GIA to the CDG beneficiary group, which is far smaller than that of the DG, which had around 1.04 million beneficiaries in September 2022. It is not clear why, despite the fact that ‘the opportunity costs are more pronounced in households with children with disabilities compared with families with adults with disabilities’ (UNICEF/DSD 2015:29), only *adults* with disabilities can access this support for human resources for care. Of course, it would be more sustainable (and likely more beneficial) to improve the availability and quality of complementary social services, rather than simply to offer another cash transfer. One intervention that would alleviate an egregious and avoidable expense would be the provision of disability-friendly transport, especially for access to health and educational services. In the Western Cape, accessible transport is available through a subsidised service, but respondents reported that the very limited vehicle fleet generally prioritises transporting working-age adults to employment or training opportunities. There is a conspicuous gap for caregivers who require the same for their children with disabilities, which limits their participation in society. However, improving these kinds of services for children with disabilities is likely to take a long time and will require a renewed commitment to important advocacy and research conducted to date. In the meantime, extending eligibility for the GIA to include CDG beneficiaries would at least facilitate some additional financial support for these families.

### Reflecting on ‘benign neglect’: Why are children with disabilities and their families always at the back of the queue?

The concept of ‘benign neglect’ originates from 1970s racialised urban planning in the United States (US). During this period, the US government responded to the deprivation and need in ‘black neighbourhoods’ by neglecting to direct additional resources and services to these areas and allowing their suffering to continue. More recently, the idea has been used to interrogate stilted progress towards racial equity in the quality of healthcare and clinical care for non-Hispanic black and Hispanic infants in the US (Rowley & Hogan 2012) and in a critique of SA’s refugee and informal sector policy implementation (Crush, Skinner & Stulgaitis 2017). McEntee-Atalianis and Vessey provide a definition of benign neglect as ‘inaction or inattention that ultimately benefits some parties and negatively impacts upon others’ (McEntee-Atalianis & Vessey 2021:4). Rowley and Hogan (2012) describe benign neglect as ‘a policy or attitude of ignoring a situation instead of assuming responsibility for managing or improving it [which] causes inaction in the face of need’ (p. 83). Borrowing from these conceptions, it seems that while it may not be malicious or intentional, the needs of children with disabilities and their families have been systematically and repeatedly deprioritised, resulting in (not so) benign neglect by the South African government. Strong rhetorical commitments exist but are not adequately supported by funding, sustained focus and mechanisms for accountability. This may be because there is a perception that it would be overly expensive to do this properly or, more cynically, perhaps the belief that a lack of well-being is inevitable for these families.

While research that focuses specifically on the CDG has been limited, patterns of deprivation and inequitable access for children with disabilities and their families, particularly those who are also living in poverty, have been reported since the early 2000s in SA. For example, accessible transport is an ongoing issue that was ‘identified in early research over two decades ago and remains largely unaddressed’ (Gibberd & Hankwebe 2022:6), an assertion these authors made based on Department of Transport reports from 1999 and 2020, respectively. Similarly, the right to education and adequate healthcare are constitutional entitlements, but because even children without disabilities are often unable to access these rights as a result of similar limitations around public transport and uneven resource distribution, the rights of children with disabilities appear to have been deprioritised (McKenzie & Chataika 2017; Modula 2022; Philpott & McKenzie 2017; Philpott & Muthukrishna 2019). In this small study, despite respondents’ sons and daughters being born between 1985 and 2013, similar narratives were shared about the difficulties related to infrastructural and systemic constraints and social exclusion. Important disability-related policy shifts have occurred in the interim (not least the transition to a democratic government), but practically, little appears to have changed for these children and their caregivers in terms of their day-to-day lives, the cost of living and the emotional strain of living in a society that does not understand or sufficiently provide for their access and participation.

The COVID-19 pandemic offers two specific illustrations of how this neglect can manifest. In 2018, SASSA officials expressed their intention to increase CDG beneficiary numbers by making people aware of the grant through ‘more effective communications’ (Kidd et al. 2018:49). There is also strong local and international evidence that children with disabilities experienced particularly intense exclusion during the pandemic (Houtrow et al. 2020; McKinney 2021; Ned, Dube & Swartz 2022; Patel 2020). Despite these indicators of willingness and need, however, recent amendments to the *Social Assistance Act* were focused on ‘topping up’ the CSG, institutionalising the Social Relief of Distress Grant (used as emergency relief during the pandemic) and setting up an inspectorate to examine fraud (South African Parliament 2020). The amended act did not single out CDG recipients. Subsequently, regulations released in May 2022 provided a valuable clarification of the definition of ‘permanent care’ and ‘support services’^1^ (DSD 2022), terms that have been central to CDG eligibility since the act’s inception in 2004 but which were never formally defined. In addition, periodic reviews of childhood disability (i.e. provision for short-term or temporary CDGs) have been included in a move away from offering only a ‘permanent’ (i.e. until the recipient child turns 18) CDG. However, these shifts appear to be more focused on limiting and gatekeeping access to the CDG, rather than on extending its reach and impact. This may be because SASSA’s primary concern appears to be that the CDG is being over-prescribed by assessing doctors and distributed to caregivers for ‘too long’, that is, beyond when a child has ‘grown out of’ their additional support needs (Trafford & Swartz 2021). No reliable data exists to document CDG exclusion errors, but in a 2017 investigation of the DG (the grant most associated with erroneous or fraudulent access), national inclusion errors were estimated at 8% lower than exclusion errors, at 34% versus 42%, respectively (Hanass-Hancock & McKenzie 2017:3). Even for the DG then, and certainly for the CDG, increasing rather than limiting access should be government’s primary concern.

With regard to the CDG specifically, there appears to be no serious investment, political or financial, from government to extend the system around the CDG or to improve how it functions. Should further amendments to the *Social Assistance Act* be made in the coming years, it is important that the focus is on enhancement and inclusion, rather than on strictly policing the boundaries of a grant that the government itself has noted is undersubscribed. The experiences of the pandemic have also revitalised older discussions about and calls for a universal basic income grant to support working-age adults who are without disabilities but are living in a context of excessively high unemployment and highly unequal service delivery (Matthews, Groenewald & Moolman 2022). These are important moves towards raising the standard and quality of life in SA, but there is also a danger that the process may, once again, overshadow the specific experiences and needs of families of children with disabilities, pushing them down to the bottom of the priority list. These grants could be complementary, so if a basic income grant (BIG) is instituted, it is recommended that CDG beneficiaries ought also to be eligible for this support, as long as they also meet BIG eligibility requirements.

### Strengths and limitations

The key strength of this work is that it adds to a limited evidence base and that this data is being disseminated rapidly. To try to mediate somewhat the limitation introduced by remote data collection, research with caregivers was delayed as long as possible and arrangements were made to speak with all caregivers who responded to recruitment calls, resulting in a relatively small and nongeneralisable sample. However, the intention of this research was not to obtain national representation but rather to gain a deeper understanding of specific feelings about the CDG, as well as what was happening in the participants’ lives and what mattered most to them. Conducting IDIs online also presented the possibility of an additional barrier between participant and researcher. This may indeed have added some distance between us, but it also meant that participants could choose to do these interviews from their own homes and at a time that suited them, rather than being limited by the times available at a rented venue. Doing these interviews at home may also have made participants *less* candid, for fear of being overheard by family. However, two of the IDI participants explicitly noted that this was valuable for them because they could be with or in the vicinity of their child, decreasing the stress of worrying how their child might be feeling or if they needed something during the interviews. Informal conversations with all participants before and after data collection also helped to build rapport and improve insight into their lives.

## Conclusion

While SA’s social assistance provisions are among the strongest when compared with regional neighbours and similar economies (Kidd et al. 2018), they are considerably weakened by the lack of attention to high-quality accessible public services (Matthews et al. 2022), particularly for the population with disabilities. As Hanass-Hancock and McKenzie (2017:9) argue, it is important to ask if SA’s disability-related grants are intended as a poverty alleviation mechanism or as one aspect of a social protection system aimed at facilitating the equal participation and improved well-being of children and adults with disabilities. A society-wide approach should be urgently initiated to emphasise disability inclusivity and to strengthen the implementation of existing guidelines and policies designed to uplift children with disabilities (Makwela & Smit 2022; Sadiki 2022). The re-education of the non-disabled public at all levels is part of this process, but it is also critical that the visibility of children and adults with disabilities is increased and the issues that are important to them are amplified (Duma et al. 2021; Swartz 2012). More opportunities should be made available for caregivers to share their narratives and have them taken seriously by government (Pitasse Fragoso 2022; Pitasse Fragoso & Lippmann 2020). South Africa has a long history of advocacy that has yielded positive change for groups oppressed because of racism, homophobia or stigmatisation as a result of association with an infectious disease. Programmes aimed at health and social workers, the public media and government officials have set a successful precedent. The country could build on this tradition to improve the inclusion of people with disabilities, throughout the life-course, beginning with children with disabilities and their families.

To close, this study echoes Dimhairo’s (2013) conclusion, made almost a decade ago, that:

[*C*]hildren with disabilities – and those caring for them – are disadvantaged in quite intricate ways and that only a more rigorous and socially sensitive design of the care dependency grant can ameliorate such disadvantage. (p. v)

Legislative changes are important, but on their own they do not represent any significant revision of a system that is functioning poorly. Interpersonal (and interdepartmental) relationships, organisational resourcing and ableism have a profound effect on policy implementation (Hoag 2010; Evans 2016; Lipsky 2010; Nothdurfter & Hermans 2018), and these dynamics must be better understood. A coherent and cohesive strategy must be designed that properly acknowledges and accounts for the actual circumstances that these caregivers are facing and goes far beyond just poverty alleviation. This shift would bring the country better into alignment with its regional and international obligations towards people with disabilities; the scaffolding is there, but there is still much work to be done. Otherwise, the CDG will continue to serve as just a stop-gap survival mechanism, insufficient on its own for meeting SA’s expressed commitment to providing a comprehensive social protection system for children with disabilities and their caregivers.

## Footnotes

1 According to these regulations, ‘“permanent care” means caring for a care dependent child … on a 24-hour basis by (a) a primary care giver; (b) a foster parent; or (c) a parent’, while ‘“support services” means [sic] (a) a day care facility; (b) a stimulation centre; (c) early childhood development services for children with disabilities; or (d) schools for learners with special education needs’ (DSD 2022:9–11).
